# Emerging Role of Non-Coding RNAs in Esophageal Squamous Cell Carcinoma

**DOI:** 10.3390/ijms21010258

**Published:** 2019-12-30

**Authors:** Qingqing Feng, Hongli Zhang, Denglin Yao, Wei-Dong Chen, Yan-Dong Wang

**Affiliations:** 1 State Key Laboratory of Chemical Resource Engineering, College of Life Science and Technology, Beijing University of Chemical Technology, Beijing 100029, Chinahlz13084@163.com (H.Z.); darrinyao@outlook.com (D.Y.); 2 Key Laboratory of Molecular Pathology, School of Basic Medical Science, Inner Mongolia Medical University, Hohhot 010110, China; 3 Key Laboratory of Receptors-Mediated Gene Regulation and Drug Discovery, School of Medicine, Henan University, Kaifeng 475004, China

**Keywords:** ncRNA, miRNAs, lncRNAs, circRNAs, ESCC

## Abstract

Esophageal squamous cell carcinoma (ESCC) is a highly prevalent tumor and is associated with ethnicity, genetics, and dietary intake. Non-coding RNAs (ncRNAs), specifically microRNAs (miRNAs), long ncRNAs (lncRNAs), and circular RNAs (circRNAs) have been reported as functional regulatory molecules involved in the development of many human cancers, including ESCC. Recently, several ncRNAs have been detected as oncogenes or tumor suppressors in ESCC progression. These ncRNAs influence the expression of specific genes or their associated signaling pathways. Moreover, interactions of ncRNAs are evident in ESCC, as miRNAs regulate the expression of lncRNAs, and further, lncRNAs and circRNAs function as miRNA sponges to compete with the endogenous RNAs. Here, we discuss and summarize the findings of recent investigations into the role of ncRNAs (miRNAs, lncRNAs, and circRNAs) in the development and progression of ESCC and how their interactions regulate ESCC development.

## 1. Introduction

Esophageal cancer (EC) is the eighth most prevalent malignant tumor and sixth leading cause of cancer-related death worldwide [1,2]. The occurrence rate of EC varies depending upon geographical location and esophageal squamous cell carcinoma (ESCC) is observed to be the most prevalent type of EC [3]. Pathogenesis of ESCC is related to ethnicity, genetics, and dietary intake [4]. Consistent with other cancers, ESCC is characterized by epigenetic abnormalities and dysregulation in the signaling pathways [3]. Chemotherapy, radio-chemotherapy, and esophagectomy are the predominant therapeutic strategies for ESCC. However, the 5-year survival rate is still poor (<15%) [5,6]. Thus, it is of high importance to understand the pathogenetic mechanisms of ESCC and develop effective strategies to treat ESCC.

Recently, non-coding RNAs (ncRNAs) have gained attention as a potential tool for treating different cancers, including ESCC [7,8]. Conventional transcriptome studies focus on protein-coding genes. However, more than 90% of the mammalian genome has been reported to be composed of ncRNAs [9]. Typically, ncRNAs can be divided into housekeeper and regulatory ncRNAs. Housekeeper ncRNAs are commonly referred to transfer RNAs, ribosomal RNAs, small cytoplasmic RNAs, and small nuclear RNAs [10]. The regulatory ncRNAs are broadly subcategorized into microRNAs (miRNAs, 18–25 nt), small interfering RNAs (siRNAs, <200 nt), piwi-interacting RNAs (piRNAs, <200 nt), and long noncoding RNAs (lncRNAs, >200 nt) [11]. Emerging studies on regulatory ncRNAs have shown their role as biomarkers or physiological regulators in many types of cancers, such as breast cancer, osteosarcoma, lung cancer, hepatocellular carcinoma, cervical cancer, bladder cancer [12,13,14,15,16]. Moreover, there have been increased investigations into the role of ncRNAs in ESCC [17,18,19]. Chen et al. revealed that lncRNA FAM201A increases radiosensitivity of ESCC by downregulating miR-101 while upregulating ataxia telangiectasia mutated (ATM) and mammalian target of rapamycin (mTOR) [20]. Thus, investigating the functions of ncRNAs and understanding their regulatory mechanism in ESCC is crucial for the further development of effective strategies to treat ESCC.

Circular RNAs are considered to be a special type of lncRNA, and display little susceptibility to exonucleases, resulting in a high stability. [21]. They were first discovered in 1976; however, they were re-established by RNA sequencing in 2012 [22,23]. Emerging publications have proposed the roles of circRNAs as biomarkers and physiological regulators in the development and progression of cancer [24]. The biological functions of circRNAs have mainly been reported as miRNA sponging and transcription regulation [25,26]. These characteristics and functions of circRNAs have contributed to its roles in the biology of human cancers, including ESCC [27,28].

**Figure 1:**
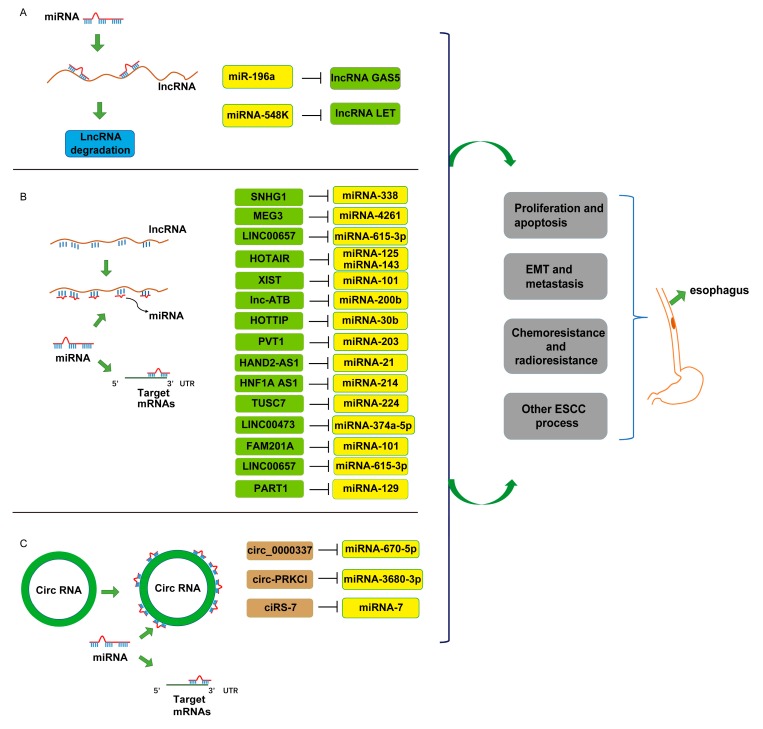
Non-coding RNA (NcRNA) regulatory interaction in esophageal squamous cell carcinoma (ESCC) progression. (**A**) MicroRNAs (miRNAs) can directly target long ncRNA (lncRNA) and regulate lncRNA expression in ESCC progression. (**B**) Long ncRNAs work as miRNA sponge to compete with endogenous RNAs. (**C**) Circular RNAs (circRNAs) release target mRNAs by competitively binding with miRNAs. These ncRNA interactions are involved in ESCC cell proliferation, apoptosis, epithelial–mesenchymal transition (EMT), metastasis, chemoradiotherapy, and other ESCC processes.

In this review, we summarize the regulatory functions of ncRNAs in ESCC. Additionally, we highlight novel functions of ncRNAs that contribute to the development of malignant phenotypes of ESCC, and how such interactions between ncRNAs influence ESCC (Figure 1).

## 2. Role of Non-Coding RNAs in ESCC Progression

To understand the role of ncRNAs in ESCC progression, numerous investigations on ncRNAs (mainly miRNAs and lncRNAs) in ESCC have been reported [7,8,29,30,31]. Many studies on the regulation of circRNAs in ESCC are in their early stages. However, there is the possibility that circRNAs may become novel potential targets for ESCC treatment. A previous report showed that Spatholobi Caulis tannin mediates several related circRNAs to suppress cell proliferation and promote apoptosis in cervical cancer [32]. In ESCC, ncRNAs have been suggested to play roles as oncogenes or tumor suppressors to regulate ESCC proliferation, apoptosis, epithelial–mesenchymal transition (EMT), metastasis, chemotherapy, or radiotherapy [33,34,35,36,37]. Additionally, several ncRNAs have been observed to serve as prognostic markers in patients with ESCC [5,38]. Several studies which suggest the role and mechanism of ncRNAs in the development of ESCC have been summarized in Table 1.

## 3. Non-Coding RNAs Regulate Cell Proliferation and Apoptosis during ESCC Development

The capability to regulate cell proliferation and apoptosis is crucial in cancer therapy. Various publications have shown that ncRNAs, such as miRNAs and lncRNAs, mediate ESCC cell proliferation and cell apoptosis or function mutually to regulate cell migration and invasion [59,64,82].

MicroRNAs control ESCC development by directly binding to mRNAs, which results in the translation repression of mRNAs [140]. During the past two years, a large number of miRNAs have been revealed to be functional regulators of cell proliferation and apoptosis (Table 1). For example, Liu et al. reported that miR-1 expression is downregulated in ESCC tissue and plasma compared to miR-1 expression in matching adjacent normal tissues. Furthermore, they found that miR-1 may inhibit ESCC development via directly targeting 3′-UTR of neurogenic locus notch homolog protein 2 (Notch2) thereby suppressing cell proliferation, migration, and invasion [59]. In addition, miRNAs have been suggested to function as mediators for other drugs to control ESCC progression. MicroRNA-30d was observed to be upregulated which may be directly suppressing PI3K regulatory subunit 2 (PIK3R2). Additionally, the effects caused by PI3K/AKT signaling inhibition, apoptosis and cell cycle arrest have been reported to have been partially restored by anti-miRNA-30d [141]. Many such studies have been published so far, which reveal miRNAs as tumor suppressors or oncogenes (Table 1). For example, miR-146a, miR-133b, miR-106b-3p, miR-219-5p, miR-206, miR-384, miR-455-5p, miR-128, miR-145-3p/5p, miR-10b-3p, miR-874-3p, miR-10a, miR-365, miR-301a, miR-6775-3p, miR-139-5p, miR-516b, miR-449a-5p, miR-125b, miR-433-3p, miR-370, miR-133b, miR-30a-3p/5p, miR-34a, miR-196a, and miR-125b-5p have been shown to act as tumor suppressors by inhibiting ESCC cell proliferation, promoting apoptosis by directly targeting oncogenes, or antagonizing pro-cancer signaling pathways [29,33,35,40,41,42,43,44,45,46,47,48,49,50,51,52,54,55,56,57,58,60,61,62,63,110,133]. In contrast, pro-oncogenes such as miR-141, miR-21, miR-10b-3p, miR-424, miR-675-3p, miR-543, miR-135, miR-23b-3p, miR-502, miR-21-5p, and miR-548k have been reported to play contrasting roles in promoting cell proliferation or suppressing apoptosis in ESCC [47,64,65,66,67,68,69,70,71,72,73]. Overall, the roles of miRNAs in cell proliferation and apoptosis can be crucial in ESCC pathogenesis.

Long ncRNAs can function as gene regulators by interacting with DNA (e.g., as promoters), RNA, or proteins [142]. Accumulating evidence has proposed that lncRNAs play a significant role in the biological development of ESCC, such as in the regulation of cell proliferation and apoptosis [7,143]. For instance, lncRNA small nucleolar host gene 1 (SNHG1), SNHG6, and SNHG16 have been reported to promote ESCC cell proliferation [74,75,76,77,78]. The lncRNA SNHG16 promotes ESCC proliferation through activating Wnt/β-catenin signaling and targeting miR-140-5p/zinc finger E-box binding homeobox 1 (ZEB1) axis [77,78]. Long ncRNA SNHG1 is upregulated in ESCC tissues and high expression of SNHG1 can be positively correlated with ESCC lymph node metastasis and decreased overall survival. Furthermore, it has been reported that silencing lncRNA SNHG1 inhibits ESCC cell proliferation and EMT capability via antagonizing Notch signaling [74]. Yan et al. have described lncRNA SNHG1 as a promoter of ESCC cell proliferation. They showed that SINHG1 directly interacts with miR-338 and competes with it, directly targeting proto-oncogene cystatin C3 and plays a role as a tumor suppressor in ESCC cells [75]. These studies demonstrate that the mechanisms of lncRNAs in ESCC are not specific. Long ncRNA colon cancer-associated transcript 1 (CCAT1) has been shown to undergo different mechanisms in nucleus and cytoplasm, and thereby promote cell growth and migration. In the nucleus of the ESCC cell, CCAT1 functions as a suppressor of sprout RTK signaling antagonist 4 (SPRY4) by linking the enhancer of zeste homolog 2 (EZH2) and the suppressor of variegation 3-9 homolog 1 (SUV39H). Inversely, CCAT1 has been shown to promote homeobox B13 (HOXB13) expression by functioning as an miR-7 sponge in the cytoplasm [31]. Thus, varying results suggest that lncRNAs may be inhibiting or promoting ESCC development by different mechanisms. The role of lncRNAs in ESCC cell proliferation and apoptosis has been extensively studied and the findings of a few relevant studies are summarized in Table 1.

As a novel category of ncRNAs, investigations on the role of circRNAs in ESCC development are still in its initial stages [18,144]. Compared with adjacent non-cancerous tissues, several circRNAs, such as circ-0067934, circ-PRKCI, circRNA-100876, circ-DLG1, and ciRS-7, have been observed to be significantly upregulated in ESCC tissues. Moreover, circ-0067934, circ-PRKCI, circRNA-100876, and circ-DLG1 have been reported to induce cell proliferation, ciRS-7 enhanced cell migration, and invasion abilities in the progression of ESCC **[18,27,28,104,105]**. Thus, the role of circRNAs in ESCC should be further investigated, which may provide novel strategies for ESCC treatment.

## 4. Non-Coding RNAs and EMT and Metastasis in ESCC

During cancer development, malignant tumors and >90% of cancer-related deaths are characterized by cells of epithelial origin, which have invaded neighboring or distant tissues and organs where they promote the formation of secondary tumors [145]. EMT involves transformation of polarized epithelial cells into mesenchymal phenotype with high motility and thus, it is an essential cellular process for tumor metastasis [146]. As a result, understanding and targeting ncRNAs to suppress EMT and tumor metastasis is a potential course of action for inhibiting the progression of cancer malignancy. 

Meng et al. showed that high expression levels of miR-6775-3p are positively correlated with good clinical outcomes. In vivo studies have shown that miR-6775-3p inhibits liver metastasis of ESCC by binding to the 3’-UTR of the melanoma antigen gene A (MAGE-A) family of tumor antigens [52]. Furthermore, miR-30c has been known to impair EMT capabilities of cells by directly targeting snail family transcriptional repressor 1 (SNAI1) in ESCC [108]. Thus, the regulatory mechanisms of miRNAs for EMT and tumor metastasis are diverse. Studies have reported that miR-34a functions as an EMT inhibitor as it reduces the intensity of ESCC progression by binding to phospholipase C elipson 1 (PLCE1) [61]. In another study on ESCC progression, overexpression of miR-34a was found to decrease the number of metastatic nodules in the liver via directly targeting CD44 [110]. At present, various miRNAs have been found to regulate EMT or tumor metastasis of ESCC. It has been reported that miR-106b-3p, miR-455-5p, and miR-128 function as tumor suppressors, and have suppressed EMT or metastasis in ESCC [33,43,44]. While miRNAs, such as miR-543, miR-23b-3p, miR-25, and miR-99b/let-7e/miR-125a play oncogenic roles in promoting EMT or metastasis during ESCC progression [68,70,109,113]. These miRNAs could serve as promising therapeutic targets in ESCC development.

Unlike miRNAs, most lncRNAs promote EMT and tumor metastasis in ESCC. The expression of LBX2-AS1 was found to be upregulated in ESCC metastatic tissues. In terms of mechanism, lncRNA LBX2-AS1 enhances ZEB1 and ZEB2 mRNA stability by interacting with RNA-binding protein heterogeneous nuclear ribonucleoprotein C (HNRNPC). Consecutively, ZEB1 may function as a transcriptional activator and activate LBX2-AS1. Due to such interactions, lncRNA LBX2-AS1 can contribute to the malignant progression of ESCC by promoting tumor migration and EMT [114]. Similarly, lncRNA-ECM, lncRNA-FTH1P3, and lncRNA-NMR have been reported to exacerbate the malignant progression of ESCC by promoting EMT or tumor metastasis [88,115,116]. Some anti-oncogenic lncRNAs such as LINC00675 and RP11-766N7.4 may inhibit EMT in ESCC [83,122]. Overall, these lncRNAs have the potential to be therapeutic targets to delay the malignant progression of ESCC by inhibiting EMT and tumor metastasis.

## 5. Non-coding RNAs Influence Chemoresistance and Radioresistance in ESCC

Among the therapeutic strategies for ESCC, chemotherapy, chemoradiotherapy, and esophagectomy are the primary treatments. However, the 5-year survival rate is observed to be still poor [147]. This poor prognosis might be due to chemoresistance and radioresistance developed by ESCC cells [36,124]. In this case, ncRNAs may increase or decrease chemoresistance and radioresistance in the treatment of ESCC [148,149].

Regulating miRNAs as a sensitive therapy is a new prospect in ESCC treatment. Studies have reported that miR-338-5p and miR-200c enhance the radiosensitivity of ESCC by inducing apoptosis and cell cycle arrest in tumor cells, respectively [36,126]. Similarly, miR-29c, miR-125a-5p, and miR-1 enhance ESCC cell sensitivity for anticancer drugs such as 5-fluorouracil, cisplatin, and gefitinib, respectively [123,124,125]. As shown in Table 1, these reported miRNAs target different molecules or signaling pathways to decrease chemoresistance and radioresistance in ESCC. Specifically, Eichelmann et al. found that overexpression as well as knockdown of miR-130a-3p and miR-148a-3p increased the sensitivity of ESCC cells towards chemotherapeutic drugs, cisplatin and 5-fluorouracil. Furthermore, the overexpression and knockdown of the two miRNAs inhibited cell migration and induced apoptosis in ESCC cells which was different from the conventional miRNA regulatory mechanism. In brief, overexpression as well as knockdown of miR-148a-3p activated p53-dependent apoptosis by inducing Bcl2-associated protein X (BAX) levels. However, it was found to differentially mediate the expression levels of pro-apoptotic Bcl2-like protein 11 (BIM) and anti-apoptotic B-cell lymphoma 2 (BCL2). Researchers observed that overexpression of miR-148a-3p leads to significant inhibitory expression of BCL2 compared to suppression of BIM. However, when miR-148a-3p was downregulated, BIM was observed to be noticeably upregulated compared to the upregulation of BCL2. Furthermore, miR-130a-3p showed similar regulatory effects for BCL2 and X-linked inhibitor of apoptosis protein (XIAP) [34]. Balancing the regulation of several target genes is critical for an efficient response of miRNAs towards chemotherapy in ESCC. These reports reveal that miRNAs participate in the decrease of chemoresistance and radioresistance in ESCC, thereby providing a novel strategy for ESCC treatment.

In ESCC, several lncRNAs function as mediators of gene expression or signaling pathways that are involved in chemoresistance and radioresistance [5,20,38,127]. LINC00473, lncRNA FAM201A, and LINC00657 impair the effect of radiotherapy by acting as sponges for miRNAs [20,38,129]. Long ncRNA TUSC7 suppresses cisplatin and 5-fluorouracil resistance in ESCC cells by inhibiting miR-224. Overexpression of LINC01419 is observed to promote GSTP1 methylation by binding to the promoter region of the GSTP1 in ESCC cells and reduce the sensitivity of ESCC cells to 5-fluorouracil [5]. Non-coding RNAs derived from donor cell cytoplasm can be transferred to recipient cells through extracellular vesicles (EVs). Chen et al. found that linc-VLDLR, when transported by the EVs, is responsible for adriamycin resistance in ESCC cells by upregulating ATP binding cassette G2 (ABCG2) in target cells [128]. Gefitinib, an ATP competitive selective EGFR tyrosine kinase inhibitor, has been investigated in ESCC clinical studies. Kang et al. showed that lncRNA PART1 and STAT1 is highly expressed in gefitinib-resistant ESCC cells. In addition, STAT1 may function as an inducer of lncRNA PART1 by binding to its promoter region. Furthermore, PART1 may act as a sponge for miR-129 to upregulate BCL2 expression and promote gefitinib resistance in ESCC. Thus the study has revealed that an exosome can carry PART1 as cargo and transport it to sensitive recipient cells which contribute to increasing gefitinib resistance. Consistency has been observed with results of clinical research that showed that the efficacy of gefitinib treatment is worse in patients with ESCC and high expression of PART1 in serum exosome [130]. These results suggest that lncRNAs can increase or decrease chemoresistance or radioresistance, and EV-mediated lncRNAs might be transmitting chemoresistance in ESCC. Thus, such investigations on lncRNAs provide the foundation for the clinical treatment of ESCC and lncRNAs may be useful as diagnostic biomarkers.

Currently, growing investigations have revealed that functions of ncRNAs in the ESCC progression such as proliferation, EMT, and resistance cannot be ignored. However, ncRNA biology therapy of ESCC is still in the preclinical stage. The pharmacodynamics and safety of ncRNA therapy in ESCC need to be further validated by clinical trials.

## 6. Experimental Approaches of Studying Non-Coding RNAs in ESCC

Identification of aberrant ncRNAs expression in ESCC tissues is of primary importance for studying the function of ncRNAs in ESCC. Quantitative real-time PCR (q-PCR) is a classic method for detecting aberrant ncRNA expression in ESCC tissues [18,52,76]. In recent years, available microarray and next generation sequencing (NGS) techniques bring researchers more complete ncRNA expression profiles between ESCC tumor and normal tissue [5,88,118]. For example, Wang et al. detected 402 upregulated and 741 downregulated lncRNAs in ESCC tumors and adjacent normal tissues from ESCC patients by NGS [150].

In vitro studies on the function of ncRNAs in ESCC are generally based on overexpression or knockdown of an ncRNA in ESCC cell lines. Overexpression plasmid or small interfering miRNA mimics and inhibitors are commonly used for transient overexpression or knockdown of miRNAs in ESCC cells [34,52,107]. RNA interference (RNAi), mainly involving siRNA and short hairpin RNA (shRNA), has been widely applied in knockdown of ncRNAs in ESCC cells [18,27,31,80]. However, the effectiveness of overexpression plasmid, siRNAs, miRNA mimics, and inhibitors is limited. After 48 h of administration, less than 1% of the siRNA remained in the cells. Short hairpin RNA is sufficient to provide continual gene knockdown effects because it can be continuously synthesized in the host cells [151]. To ascertain the role of LINC01419 in ESCC, Chen et al. performed a knockdown of LINC01419 expression in ESCC cells using shRNA in vitro and in vivo. In xenograft models of ESCC, lentiviral vectors are common used for delivery of exogenous DNA because of their high transfection efficiency and effective integration [31,151,152]. A xenograft model can be established by direct injection of stably transfected ESCC cell lines into recipient mice. For example, Liang et al. identified the contributory role of LncRNA CASC9 in ESCC metastasis by transfecting CASC9 siRNAs or overexpression plasmid of CASC9 in vitro. In an in vivo study, KYSE150 cells stably infected with CASC9 siRNA lentiviral vector (LV-CASC9) were given to BALB/c mice by a tail vein injection [153]. At present, siRNA and shRNA still have certain off-target effects and safety problems and lentivirus vector also has certain safety and immunogenicity risks [151]. Therefore, the development and optimization of safe and effective ncRNA interference technology and delivery systems may benefit for research into the functions of ncRNAs in ESCC.

## 7. Prospects

Over a decade of research has improved our understanding of ncRNAs, from transcriptional noises to functional regulatory molecules that mediate diverse physiological and pathological processes [142,154]. Investigating the functions and mechanisms of ncRNAs provides potential therapeutic targets in treating many cancers. However, research about circRNAs regulating ESCC is in its initial stage and some mechanisms by which lncRNAs and circRNAs mediate ESCC pathogenesis is poorly known. In conclusion, ncRNAs contribute to the development and prognosis of ESCC. Further investigation of the detailed mechanisms by which ncRNAs regulate ESCC may provide new insights into how ncRNAs promote or inhibit tumor development and provide potential therapeutic targets for treating ESCC.

## Figures and Tables

**Table 1:** Regulatory non-coding RNAs (ncRNAs) in ESCC progression.

Role of ncRNAs in ESCC	ncRNAs	Identified TARGETS or Signaling Pathways	Role	Reference
Cell proliferation and apoptosis	miRNA:			
	miRNA-146a	IRS2	−	[29]
	miRNA-133b	TAGLN2	−	[39]
	miRNA-106b-3p	ZNRF3	−	[33]
	miRNA−219-5p	CCNA2	−	[40]
	miRNA-206	c-MET	−	[41]
	miRNA-384	LIMK1	−	[42]
	miRNA-455-5p	Rab31	−	[43]
	miRNA-128	COX2	−	[44]
	miRNA 145 3p, 5p	DHRS2 and MYO1B, Sp1	−	[45,46]
	miRNA-10b-3p	FOXO3	−	[47]
	miRNA-874-3p	STAT3	−	[48]
	miRNA-10a	Tiam1	−	[49]
	miRNA-365	PSAT1	−	[50]
	miRNA-301a	WNT1	−	[51]
	miRNA-6775-3p	MAGE-A and SLC7A5	−	[52]
	miRNA-139-5p	VEGFR	−	[53]
	miRNA-516b	CCNG1	−	[54]
	miRNA-449a-5p	Cyclin D1	−	[55]
	miRNA-125b	BMF	−	[56]
	miRNA-433-3p	GRB2	−	[57]
	miRNA-370	PIN1	−	[58]
	miRNA-133b	Cullin 4B	−	[35]
	miRNA-1	Notch2	−	[59]
	miRNA-30a-3p, 5p	Wnt2, FZD2	−	[60]
	miRNA-34a	PLCE1	−	[61]
	miRNA-196a	lncRNA GAS5	−	[62]
	miRNA-125b-5p	HMGA2	−	[63]
	miRNA-141	YAP1 and SOX17	+	[64]
	miRNA-21	RASA1	+	[65]
	miRNA-424	PRKCD and WEE1	+	[66]
	miRNA-675-3p	NA	+	[67]
	miRNA-543	PLA2G4A	+	[68]
	miRNA-135	RERG	+	[69]
	miRNA-23b-3p	EBF3	+	[70]
	miRNA-502	NA	+	[71]
	miRNA-21-5p	CADM2	+	[72]
	miRNA-548k	lncRNA-LET	+	[73]
	LncRNAs:			
	LncRNA SNHG1	miRNA-338/CST3,Notch signaling	+	[74,75]
	LncRNA SNHG6	NA	+	[76]
	LncRNA SNHG16	Wnt/β-catenin,miRNA-140-5p/ZEB	+	[77,78]
	LncRNA MEG3	miRNA-4261	+	[79]
	LINC01980	GADD45A	+	[80]
	FMR1-AS1 (female patients)	TLR7	+	[81]
	DLX6AS1	NA	+	[82]
	LINC00657	miRNA-615-3p	+	[83]
	DUXAP10	p21	+	[84]
	LINC01296	NA	+	[85]
	LncRNA DANCR	NA	+	[86]
	LncRNA SOX2OT	NA	+	[87]
	LncRNA NMR	BPTF/ ERK1/2 pathway	+	[88]
	MIR31HG	NA	+	[89]
	AK001796	p53	+	[90]
	LINC01503	EBP1 and DUSP6	+	[91]
	LUCAT1	DNMT1	+	[92]
	Linc ROR	SOX9	+	[93]
	HOTAIR	miRNA-125 and miRNA-143	+	[94]
	XIST	miRNA-101/EZH2	+	[95]
	LncRNA GHET1	EMT	+	[96]
	Lnc-ATB	miRNA-200b/Kindlin-2	+	[97]
	HOTTIP	miRNA-30b/snail1, HOXA13	+	[98]
	PVT1	miRNA-203/LASP1	+	[99]
	AFAP1-AS1	NA	+	[37]
	LINC00675	Wnt/β-catenin	−	[83]
	HAND2-AS1	miRNA-21	−	[100]
	LncRNA NEF	Wnt/β-catenin pathway	−	[101]
	LncRNA GAS5	PI3K/AKT/mTOR	−	[102]
	FER1L4	NA	−	[103]
	CircRNAs:			
	Circ_0000337	miRNA-670-5p	+	[18]
	Circ-PRKCI	miRNA-3680-3p	+	[27]
	CircRNA_100876	NA	+	[28]
	Circ-DLG1	miRNAs	+	[104]
	CiRS-7	miRNA-7/KLF4 and NF-κB signals	+	[105]
	Circ_0067934	NA	+	[106]
Cell EMT and metastasis	miRNA:			
	miRNA-106b-3p	ZNRF3	−	[33]
	miRNA-455-5p	Rab31	−	[43]
	miRNA-128	COX 2	−	[44]
	miR-128-3p	ZEB1	−	[107]
	miRNA-10a	Tiam1	−	[49]
	miRNA-6775-3p	MAGE-A and SLC7A5	−	[52]
	miRNA-139-5p	VEGFR	−	[53]
	miRNA-30c	SNAI1	−	[108]
	miRNA-25	NA	−	[109]
	miRNA-34a	PLCE1, CD44	−	[61,110]
	miRNA-31	LATS2	−	[111]
	miRNA-145-5p	Sp1	−	[45]
	miRNA-377	CD133 and VEGF	−	[112]
	miRNA-543	PLA2G4A	+	[68]
	miRNA-23b-3p	EBF3	+	[70]
	miRNA-25	FBXW7	+	[109]
	miR-99b/let-7e/miR-125a	ARID3A	+	[113]
	LncRNA:			
	LBX2-AS1	ZEB1 and ZEB2	+	[114]
	LncRNA-ECM	ICAMI	+	[115]
	FTH1P3	SP1/NF-kB	+	[116]
	NMR	BPTF/ ERK1/2 pathway	+	[88]
	Linc-UBC1	EZH2 and E-cadherin	+	[117]
	CASC9	CBP and LAMC2	+	[118]
	LincRNA-ROR	miR-145/FSCN1	+	[119]
	SNHG16	miR-140-5p/ZEB1	+	[77]
	LncRNA SNHG1	Notch signaling	+	[74]
	LncRNA GHET1	EMT	+	[96]
	HNF1A-AS1	miR 214/SOX-4	+	[120]
	Lnc-ATB	miR-200b/Kindlin-2	+	[97]
	HOTTIP	WDR5 and HOXA13	+	[98,121]
	LINC00675	Wnt/β-catenin	−	[83]
	RP11-766N7.4	NA	−	[122]
Chemosensitivity and radiosensitivity	miRNA:		−	
	miRNA-29c (5-fluorouracil)	F-box only protein 31	−	[123]
	miR-130a-3p and miR-148a-3 (Cisplatin and 5-FU)	Bcl-2/Bim and Bcl2/XIAP	−	[34]
	miRNA-125a-5p (cisplatin)	STAT3 signaling	−	[124]
	miRNA-1 (gefitinib)	PIK3CA signaling	−	[125]
	miRNA-338-5p (radiosensitivity)	Apoptosis signaling	−	[36]
	miRNA-200c (radiosensitivity)	Cell cycle arrest and p21	−	[126]
	LncRNA TUSC7 (cisplatin or 5-Fu)	miRNA-224/DESC1	−	[127]
	LINC01419 (5-fluorouracil)	GSTP1 methylation	+	[5]
	LINC00473 (radiotherapy)	miRNA-374a-5p	+	[38]
	Linc-VLDLR in extracellular vesicles (adriamycin)	NA	+	[128]
	LnRNA FAM201A (radiotherapy)	miRNA-101/ATM and mTOR	+	[20]
	LINC00657 (radiotherapy)	miRNA-615-3p and JunB	+	[129]
	LncRNA PART1 (gefitinib)	miRNA-129/Bcl-2 pathway	+	[130]
	MALAT1 (radiotherapy)	Cks1	+	[131]
Prognostic biomarkers	miRNA:			
	miRNA-506, miRNA-145, miRNA-375, miRNA-655, miRNA-874-3p, miRNA-9			[48,132,133,134,135]
	LncRNA:			
	MEG3, SEMA3B-AS1, SNHG6, AK001796, ANRIL, BANCR, UCA1 and MALAT1, MIR31HG, FOXD2-AS1, LINC01296			[76,79,85,89,90,136,137,138,139]

+, Oncogene; −, a tumor suppressor; NA, not available. EMT, epithelial–mesenchymal transition; IRS2, insulin receptor substrate 2; TAGLN2, transgelin-2; ZNRF3, zinc and ring finger 3; CCNA2, cyclin A2; LIMK1, LIM domain kinase 1; DHRS2, dehydrogenase/reductase member 2; MYO1B, myosin IB; Tiam1, T-lymphoma invasion and metastasis protein inducing protein 1; PSAT1, phosphoserine aminotransferase 1; SLC7A5, solute carrier family 7 member 5; BMF, BCL-2-modifying factor; GRB2, growth factor receptor-bound protein 2; PIN1, peptidyl-prolyl cis-trans isomerase NIMA-interacting 1; PLCE1, phospholipase C elipson 1; HMGA2, high mobility group protein A2; RASA1, RAS p21 protein activator 1; YAP1, yes- associated protein 1; SOX17, SRY-box 17; PLA2G4A, phospholipase A2 group IVA; RERG, RAS like estrogen regulated growth inhibitor; GADD45A, DNA damage inducible 45 alpha; BPTF, bromodomain PHD finger transcription factor; DNMT1, DNA methyltransferase 1; PLCE1, phospholipase C elipson 1; LATS2, large tumor suppressor kinase 2; ARID3A, AT-rich interaction domain 3A; FBXW7, F-box and WD repeat domain-containing 7; BPTF, bromodomain PHD finger transcription factor.
